# Rural-urban Differences in Long-term Mortality and Readmission Following COVID-19 Hospitalization, 2020 to 2023

**DOI:** 10.1093/ofid/ofae197

**Published:** 2024-04-05

**Authors:** Mohammed Yousufuddin, Maryam Mahmood, Ebrahim Barkoudah, Fatimazahra Badr, Kanika Khandelwal, Warren Manyara, Umesh Sharma, Ahmed D Abdalrhim, Meltiady Issa, Sumit Bhagra, Mohammad H Murad

**Affiliations:** Department of Hospital Internal Medicine, Mayo Clinic Health System, Austin, Minnesota, USA; Division of Public Health, Infectious Diseases, and Occupational Medicine, Mayo Clinic, Rochester, Minnesota, USA; Department of Internal Medicine/Hospital Medicine, Brigham and Women's Hospital, Boston, Massachusetts, USA; Department of Hospital Internal Medicine, Mayo Clinic Health System, Austin, Minnesota, USA; Department of Hospital Internal Medicine, Mayo Clinic Health System, Austin, Minnesota, USA; Department of Hospital Internal Medicine, Mayo Clinic Health System, Austin, Minnesota, USA; Division of Hospital Internal Medicine, Mayo Clinic, Phoenix, Arizona, USA; Division of General Internal Medicine, Mayo Clinic, Rochester, Minnesota, USA; Division of Hospital Internal Medicine, Mayo Clinic, Rochester, Minnesota, USA; Department of Endocrine and Metabolism, Mayo Clinic Health System, Austin, Minnesota, USA; Division of Public Health, Infectious Diseases, and Occupational Medicine, Mayo Clinic, Rochester, Minnesota, USA

**Keywords:** and mortality, COVID-19, readmission, rurality

## Abstract

**Background:**

We compared long-term mortality and readmission rates after COVID-19 hospitalization based on rural-urban status and assessed the impact of COVID-19 vaccination introduction on clinical outcomes by rurality.

**Methods:**

The study comprised adults hospitalized for COVID-19 at 17 hospitals in 4 US states between March 2020 and July 2022, followed until May 2023. The main analysis included all patients, whereas a sensitivity analysis focused on residents from 4 states containing 17 hospitals. Additional analyses compared the pre- and postvaccination periods.

**Results:**

The main analysis involved 9325 COVID-19 hospitalized patients: 31% were from 187 rural counties in 31 states; 69% from 234 urban counties in 44 states; the mean age was 65 years (rural, 66 years; urban, 64 years); 3894 women (rural, 41%; urban, 42%); 8007 Whites (rural, 87%; urban, 83%); 1738 deaths (rural, 21%; urban, 17%); and 2729 readmissions (rural, 30%; urban, 29%). During a median follow-up of 602 days, rural residence was associated with a 22% higher all-cause mortality (log-rank, *P* < .001; hazard ratio, 1.22; 95% confidence interval, 1.10-1.34, *P* < .001), and a trend toward a higher readmission rate (log-rank, *P* = .038; hazard ratio, 1.06; 95% confidence interval, .98-1.15; *P* = .130). The results remained consistent in the sensitivity analysis and in both pre- and postvaccination time periods.

**Conclusions and Relevance:**

Patients from rural counties experienced higher mortality and tended to be readmitted more frequently following COVID-19 hospitalization over the long term compared with those from urban counties, a difference that remained even after the introduction of COVID-19 vaccines.

In 2020, COVID-19 emerged as the third leading cause of mortality in the United States, with high death rates in urban areas at the beginning of the pandemic [1]. By December 2020, the COVID-19 mortality pattern changed with higher mortality in rural areas [1, 2]. Epidemiological studies showed that rural populations were at a higher risk of serious COVID-19-related illness with high rates of hospitalizations [3]. The scarcity of reports raised doubts about whether the observed disparity in COVID-19 mortality between rural and urban communities in outpatient settings carried over to patients hospitalized for COVID-19. Previous studies demonstrated that approximately 9% of symptomatic patients who tested positive for SARS-CoV-2 in the community were hospitalized [4–6]. Less is known about the long-term outcomes following COVID-19 hospitalization and the impact of rurality because prior research on mortality and readmission following COVID-19 hospitalization was largely focused on in-hospital outcomes and follow-up periods ranging from 30 days to 1 year, with most research conducted on patients hospitalized during the first year of the COVID-19 pandemic [7–10].

Understanding rural-urban differences in long-term outcomes following COVID-19 hospitalization is crucial for developing sustained strategies to promote health equity for COVID-19 admissions in the ongoing pandemic. Therefore, this study primarily aimed to assess long-term all-cause mortality and all-cause readmission after hospitalization for the first occurrence of COVID-19 to determine potential disparities across the rural-urban continuum. Our secondary aims were to (1) conduct a sensitivity analysis limiting patients from the US states where the study centers were located and (2) undertake subgroup analyses across age, sex, race, marital status, pandemic year, and receipt of Emergency Use Authorized (EUA) medications.

## METHODS

### Patient Consent Statement

This research is a retrospective investigation using the review of electronic medical records for data collection, which are accessible within the records. It is classified as a low-risk study because there is no direct physical or verbal contact with the patients. Consequently, the institutional review board waived the requirement for written consent because of the minimal risk involved. The study design was approved by the Mayo Clinic institutional review board (ID: 22-007448).

### Study Design and Population

In this retrospective study, we analyzed data from COVID-19 patients hospitalized at 17 hospitals in Arizona, Florida, Minnesota, and Wisconsin, from 1 March 2020, to 22 July 2022, with follow-up until 23 May 2023. Data abstraction has been previously described [11]. The data were abstracted using the *International Classification of Disease Clinical Modification Tenth Revision (ICD-10-CM)* code U07.1 for COVID-19. Subsequently, a manual review of electronic medical records (EMR) was conducted to verify diagnoses and relevant data points. Adults aged ≥18 years with reverse polymerase chain reaction-confirmed COVID-19 were included. The following patients were excluded: (1) pregnant women, (2) patients with COVID-19 as a secondary diagnosis, (3) those with recurrent COVID-19, and (4) those who opted out from EMR-based research. The study was conducted per the declaration of Helsinki and the Strengthening of The Reporting of Observational Studies in Epidemiology (STROBE) [12].

We divided the study cohort into 2 groups for analysis: the main analysis cohort and the sensitivity analysis cohort. The main analysis cohort included all consecutive patients without consideration of their self-reported residential addresses at admission. On the other hand, the sensitivity analysis cohort consisted of a subset of patients whose addresses at admission were exclusively in Arizona, Florida, Minnesota, and Wisconsin, excluding those from other states. This division was primarily aimed at addressing potential challenges in following up with patients from states outside the locations of the 17 hospitals.

During the study period, shifts occurred in SARS-CoV-2, patient characteristics, treatment patterns, public perception, and health care policies, with the initiation of COVID-19 vaccination in the United States on 22 December 2020, being the most significant event. Because of the lack of vaccination data for most patients, we aimed to assess the impact of vaccine introduction. Thus, to assess the clinical outcomes by rurality before and after the introduction of COVID-19 vaccine, we split the patients into 2 time frames: prevaccination (1 March-21 December 2020) and postvaccination (22 December 2020-22 July 2022).

### Objectives

The study's main objective was to compare long-term mortality and hospital readmission rates between rural and urban residents following their first hospitalization for COVID-19. Secondary objectives included: (1) investigating differences in mortality and readmission rates across important clinical subgroups, (2) conducting a sensitivity analysis with patients from 4 US states, where study centers were located, while excluding residents from other states, and (3) evaluating the impact of introducing COVID-19 vaccination in the United States during the study period.

### Covariates

We collected data on demographics, marital status, current smoking, county of residence and state, height, and weight, underlying chronic conditions, admission service, and EUA COVID-19-specific or repurposed treatment. Note that medications such as remdesivir (granted EUA by the US Food and Drug Administration [FDA] on 1 May 2020), dexamethasone (not formally approved but recommended by the National Institutes of Health for COVID-19 treatment following the RECOVERY trial publication on 22 June 2020), and inhibitors of Janus kinase (JAK) and interleukin (IL) such as baricitinib (granted EUA by the FDA on 19 November 2020) and tocilizumab (granted EUA by the FDA on 24 June 2021) received EUA from the FDA for use in COVID-19 at various stages of the pandemic. Supplementary Table 1 summarizes the covariates for multivariate analysis.

### Comorbidities

The underlying comorbidities with potential impact on COVID-19 outcomes were a priori selection based on previous outcome studies by the study investigators [11], review of published literature, expert opinion, and conditions specified by the Department of Health and Human Services [13]. Supplementary Table 2 summarizes individual chronic conditions and their respective ICD-10-CM codes.

### Rural-urban Classification Scheme

We used the 2013 National Center for Health Statistics’ Urban-Rural Classification Scheme for Counties based on the Office of Management and Budget's to assign counties to 1 of the 6 levels of urbanization: 4 metropolitan (large central metropolitan, large fringe metropolitan, medium metropolitan, and small metropolitan) and 2 nonmetropolitan (micropolitan and noncore) [14]. In this study, rural refers to 2 nonmetropolitan areas and urban refers to 4 metropolitan areas.

### Mortality

We assessed all-cause deaths from admission to the censoring date, 23 May 2023. Mortality data were obtained from EMR and other sources. The Mayo Clinic updates mortality data from routine clinical care across hospitals, the network of clinics, local obituaries, and the Department of Vitals and Health Statistics.

### Readmissions

Readmission following COVID-19 hospitalization was defined as all-cause hospitalization during the study period. Patients with multiple readmissions, the first readmission was included in analysis.

### Outcomes

The primary outcomes were all-cause mortality and all-cause first readmission during the follow-up period. Each patient was followed-up from the time of admission until death or censoring.

### Statistical Analysis

We used SAS version 9.4 (SAS Institute Inc., Cary, NC) and R version 4.3.1 (R Project for Statistical Computing; R Foundation) for data analysis. Student *t* test, Kruskal-Wallis, and Pearson χ^2^ tests were used to compare baseline characteristics. We used multivariable logistic regression models adjusted for age, sex, race, and marital status to estimate odds ratios (OR) and 95% confidence intervals (CIs) for each comorbidity or the receipt of EUA drugs to compare rural and urban patients. We generated Kaplan-Meier curves to estimate the risk of death or readmission after hospitalization, stratified by rurality. Cox proportional hazard models were constructed to estimate hazard ratios (HR) and corresponding 95% CI for mortality or readmission. Collinearity among independent variables was assessed using Pearson correlation to determine whether individual variables in the multivariable models were correlated. We estimated variance inflation factor (VIF) for each covariate. We conducted sensitivity analysis in a restricted cohort limited to the US states with the study centers. Sensitivity analyses in a restricted cohort were conducted to assess whether differences in populations in the 4 US states where the study centers were located from the remaining states with no study centers could lead to differences in outcomes. Identical statistical methods were used both for main and sensitivity analysis and results were presented separately.

#### Subgroup Analysis

Cox proportional models were constructed separately to determine the association between residential status and mortality or readmission across the following prespecified subgroups of clinical importance: age (<65 years and ≥65 years), sex (female and male), race (White and non-White), marital status (married and nonmarried); COVID-19 pandemic year (2020, 2021, and 2022), treated versus untreated patients with EUA medications (remdesivir, dexamethasone, or JAK1/2 inhibitors/IL-6 inhibitor). Forest plots were generated to display point estimates with 95% CIs. Additional subgroup analyses were conducted to examine the association between residential status and mortality or readmission rates in the prevaccination and postvaccination periods.

## RESULTS

### Study Population

**Figure 1.**
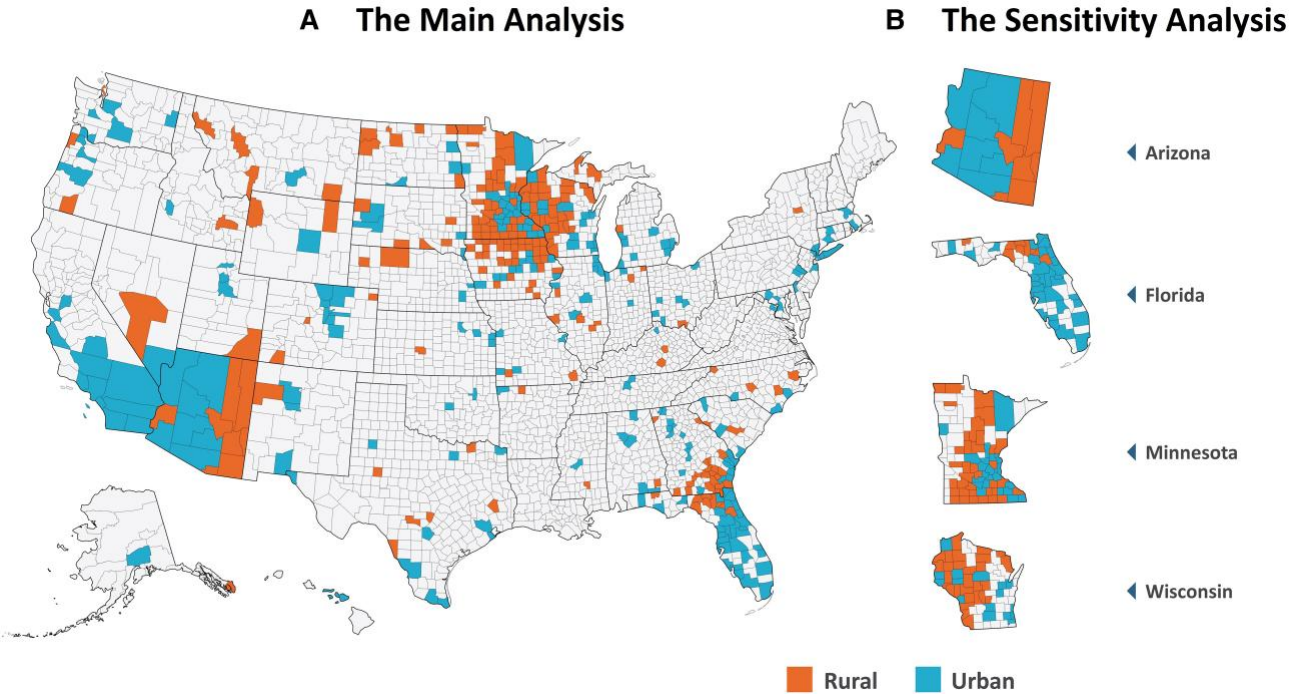
The US county map: *A*, Main analysis; *B*, sensitivity analysis. In the main analysis, the showcased counties (n = 421) represent inhabitancy of study population before hospital admission. In the sensitivity analysis, the featured counties (n = 187) represent inhabitancy of study population before hospital admission in states of Arizona, Florida, Minnesota, and Wisconsin.

The main analysis included 9325 patients (421 counties in 44 states) who were hospitalized with COVID-19. The counties included 34 large central metropolitan areas (28.5% of patients; n = 2661/9325), 67 large fringe metropolitan areas (10.2% of patients; n = 953/9325), 62 medium metropolitan areas (1.8% of patients; n = 176/9325), 71 small metropolitan areas (28.3% of patients; n = 2643/9325), 79 micropolitan (15.8% of patients; n = 1473/9325), and 108 noncore (15.2% of patients; n = 1419/9325). A STROBE flow diagram depicting cohort selection process is shown in Supplementary Figure 1. In total, 2898 (31.1%) patients originated from 187 rural counties, and 6427 (68.9%) patients came from 234 urban counties. Figure 1*A* displays a map of all US counties, with rural (red) and urban (blue) counties marked to indicate the residential areas of the study population at the time of admission in the main analysis. Figure 1*B* illustrates 4 US states (Arizona, Florida, Minnesota, Wisconsin) with counties colored red (rural) and blue (urban), representing the patients’ home counties at the time of hospitalization in the sensitivity analysis. The baseline characteristics of the main and sensitivity cohorts according to rurality are presented in Table 1. Patients from rural counties were more likely to be older, White, nonmarried (living alone), and current smokers compared to those from urban counties. The ORs and 95% CIs for the prevalence of comorbidities, adjusted for age, sex, and race, among rural and urban patients are presented in Supplementary Figure 2 for the main cohort and Supplementary Figure 3 for sensitivity analysis. The odds of the prevalence of obesity, heart failure, chronic obstruction pulmonary disease, major depression, or substance use disorder were significantly higher in adults living in rural areas than in those residing in urban areas after adjusting for age, sex, race, and marital status.

**Table 1.** Differences in Baseline Characteristics by Rural and Urban Residential Status in Main Cohort and Restricted Cohort

Cohorts	Categories	Characteristics	All Participants N (%)	Rural N = (%)	Urban N = (%)	*P* Value
**Main (N = 9325)**	Main cohort	…	9325	2898 (31)	6427 (69)	
	Demographics	Age, mean (SD), y	65 (16)	66 (16)	65 (16)	.0184
		Male	5431 (58)	1682 (58)	3749 (58)	.8029
		White	8007 (86)	2521 (87)	5486 (85)	.0369
	Social indicators	Married	5430 (58)	1634 (56)	3796 (59)	.0162
		Current smoking	584 (6)	205 (7)	379 (6)	.0336
	Calendar year	2020	2961 (32)	897 (31)	2064 (32)	.2690
		2021	4785 (51)	1533 (53)	32 582 (51)	.0417
		2022	1579 (17)	468 (16)	1111 (17)	.1795
**Restricted (N = 8757)**	Sensitivity analysis cohort	…	8757	2607 (30)	6150 (70)	
	Demographics	Age, mean (SD), years	65 (17)	66 (16)	65 (17)	.0244
		Female	5093 (58)	1091 (42)	2573 (42)	.9921
		White	7516 (86)	2267 (87)	5249 (85)	.0519
	Social indicators	Married	5021 (57)	1419 (54)	3602 (59)	.0004
		Current smoking	558 (6)	191 (7)	367 (6)	.0190
	Calendar year	2020	2751 (31)	779 (30)	1972 (32)	.0441
		2021	4513 (52)	1399 (54)	3114 (51)	.0101
		2022	1493 (17)	429 (16)	1064 (17)	.3512

Abbreviation: SD, standard deviation.

### Treatment Patterns

Patients from rural counties were more likely to receive EUA COVID-19-specific or repurposed drugs, including remdesivir (61% vs 56%), dexamethasone (62% vs 59%), and JAK1/2 inhibitors/IL-6 inhibitors (17% vs 15%) as illustrated in Supplementary Figures 2 and 3 for the main and sensitivity analyses, respectively.

### Collinearity

Collinearity matrix for independent predictor variables is presented in Supplementary Table 3 and the corresponding values for the VIF are displayed in Supplementary Table 4. The values of the VIF ranged from 1.052 to 1.464, indicating no significant correlations among predictor variables.

### Outcomes

#### Main Analysis

**Figure 2.**
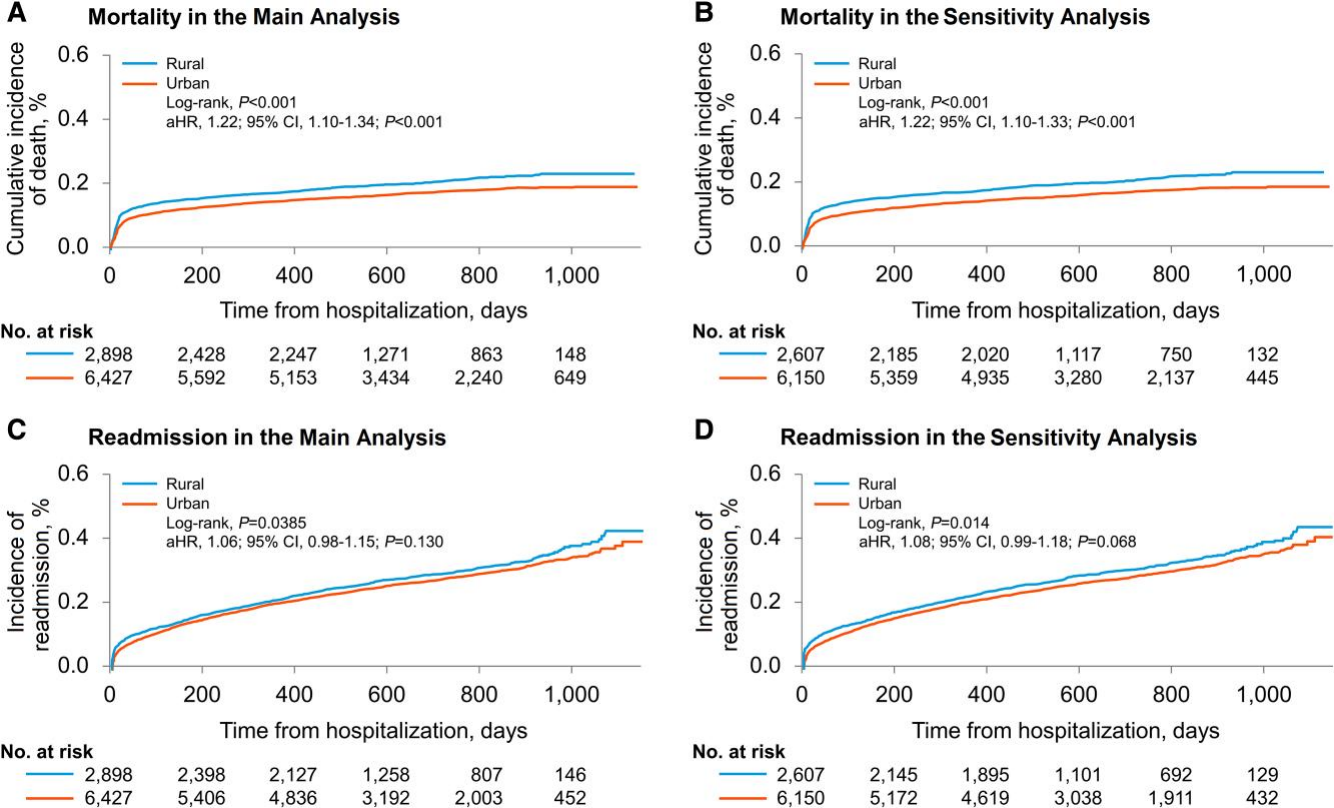
Kaplan-Meier estimates for mortality and readmission following COVID-19 hospitalization stratified by rural and urban county of residence. *A*, Main cohort: Kaplan-Meier estimates for mortality rates, categorized according to whether patients reside in rural or urban areas. *B*, Sensitivity cohort: Kaplan-Meier estimates for mortality rates, categorized according to whether patients reside in rural or urban areas. *C*, Main cohort: Kaplan-Meier estimates for readmission rates, categorized according to whether patients reside in rural or urban areas. *D*, Sensitivity cohort: Kaplan-Meier estimates for readmission rates, categorized according to whether patients reside in rural or urban areas. Abbreviations: aHR, adjusted to hazard ratio; CI, confidence interval.

**Figure 3.**
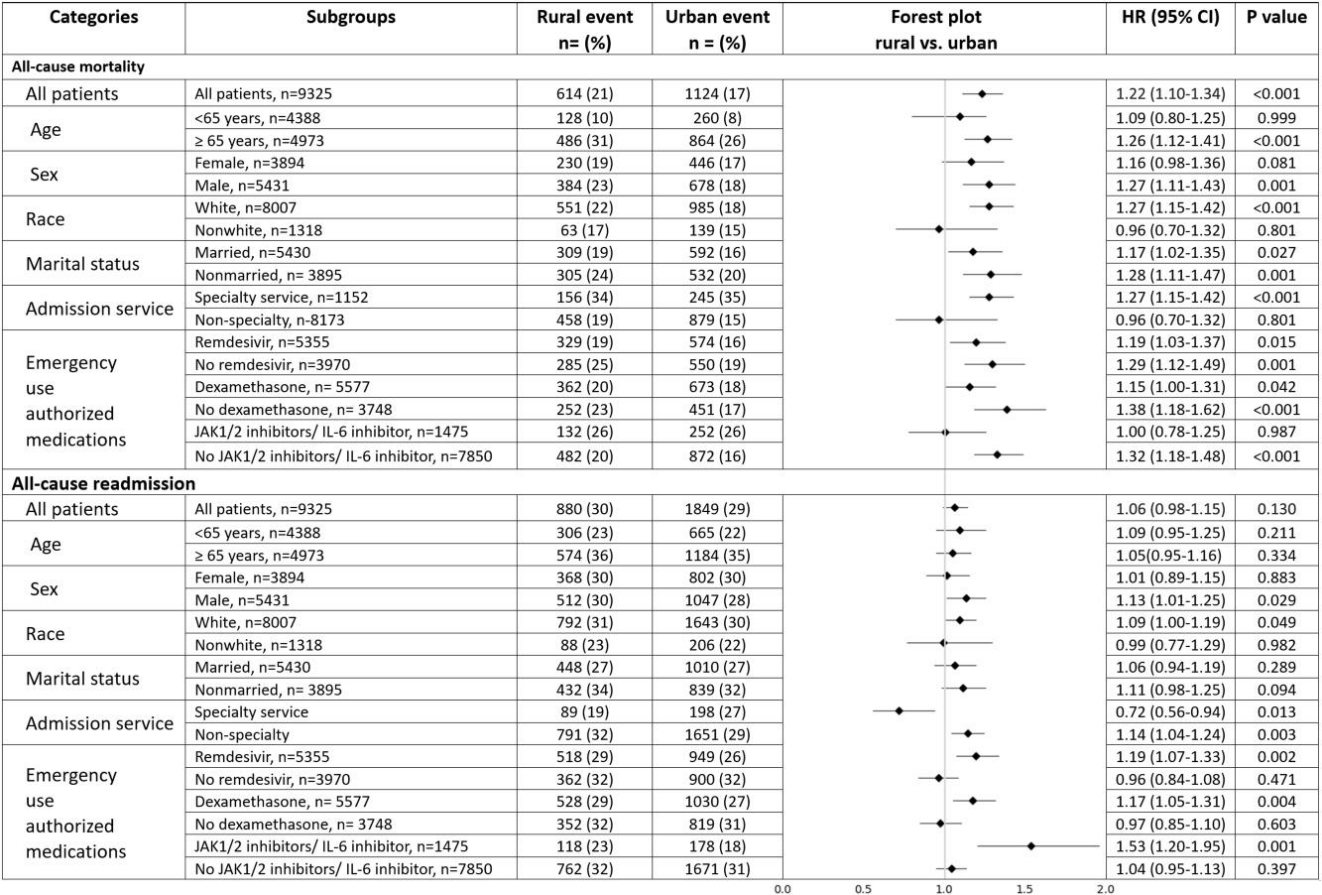
Subgroup analysis using Cox proportional hazard models. Hazard ratios (HR) and 95% confidence intervals (CIs) for long-term mortality and readmission associated with hospitalization for COVID-19 among rural versus those from urban counties. Cox proportional hazard models were adjusted for age, sex, race, marital status, cigarette smoking, hypertension, coronary artery disease, heart failure, atrial fibrillation, stroke, asthma, chronic obstructive pulmonary disease, dementia, chronic liver disease, anemia, osteoarthritis, cancer, transplant recipiency, depression, other psychiatric conditions, admission to specialty service, use of remdesivir, dexamethasone, Janus kinase (JAK) 1/2 inhibitors/interleukin (IL)-6 inhibitor in hospital, and year of the pandemic.

Patients were followed from day 0 (the day of admission) through death or censoring, with a median follow-up of 602 days (interquartile range [IQR], 462-856 days) including 574 days (IQR, 451-843 days) for rural and 619 days (IQR, 465-858 days) for urban patients. Between 1 March 2020 and 23 May 2023, the cumulative deaths from all causes were 19% (n = 1738/9325), including 21% among rural and 17% in urban patients. Figure 2 illustrates Kaplan-Meier estimates for mortality and readmission stratified by rural and urban residential status. The median time to death was 30 days (IQR, 13-198) and lower in rural than in urban patients (24 days [IQR, 12-167] in rural vs 34 [IQR, 13-208] days in urban areas, *P* = .05). Rural, compared with urban patients, had higher mortality (log-rank <.001), with absolute difference of 4%, corresponding to 40 additional deaths per 1000 rural patients. This translates to the occurrence of 1 additional death for every 25 patients admitted with COVID-19 from rural counties. Cox proportional hazard model adjusted for 31 covariates (Supplementary Table 1) demonstrated a 1.22-fold increase in mortality in patients from rural compared with those from urban counties (log-rank, *P* < .001; HR, 1.22; 95% CI, 1.10-1.34; *P* < .001). As shown in Figure 3, age ≥ 65 years (HR, 1.26; 95% CI, 1.12-1.41; *P* < .001), White race (HR, 1.27; 95% CI, 1.15-1.42; *P* < .001), admission to specialty service (HR, 1.27; 95% CI, 1.15-1.42; *P* < .001) and not receiving JAK1/2 inhibitors/IL-6 inhibitor therapy (HR, 1.32; 95% CI, 1.18-1.48; *P* < .001) were independently associated with a higher mortality among rural versus urban patients. The rural-urban difference in mortality remained consistent when analyzed by sex, marital statuses, pandemic year, admission service, and use of remdesivir and dexamethasone. The rural-urban disparity in mortality persisted in sensitivity analysis (n = 8757; log-rank *P* < .001; HR, 1.22; 95% CI, 1.10-1.33, *P* < .001).

Among the survivors of the initial COVID-19 hospitalization, 1849 (29%) and 880 (30%) patients from rural and urban counties, respectively, experienced at least 1 all-cause 1st readmission over a median follow-up of 602 days. The median time to readmission was 155 (IQR, 19-392) days, which was shorter in rural than in urban patients (152 [IQR, 12-389] days in rural areas; 157 [IQR, 24-399] days in urban areas, *P* = .027). Although, Kaplan-Meier estimates showed a significant rural-urban difference in readmission rate (log-rank, *P* = .038), Cox regression analysis, which accounted for 31 covariates, showed a trend toward higher readmission rates (HR, 1.06; 95% CI, .98-1.15; *P* = .130).

#### Sensitivity Analysis

The sensitivity analysis consisted of 8757 (rural, n = 2607 [30%]; urban, 6150 [70%]) patients of 164 counties (rural, 81; urban, 83) in 4 US states. The adjusted ORs and 95% CIs for the prevalence of comorbidities among rural and urban patients are presented in Supplementary Figure 3. The cumulative deaths from all causes were 19% (n = 1626/8787), including 21% among rural and 17% in urban patients. Similarly, the all-cause readmission rate was 30% (n = 2597/8787), including 31% among rural and 29% in urban patients. In the sensitivity cohort, both mortality (log-rank *P* < .001; HR, 1.22; 95% CI, 1.10-1.33; *P* < .001) and readmission rates (log-rank, *P* = .014; HR, 1.08; 95% CI, .99-1.18; *P* = .068) were consistent with those observed in the main cohort. The results of the sensitivity analysis are presented in Table 1 and Supplementary Figures 3 and 4.

#### Pre- and Postvaccination Timeframe Analysis

**Figure 4.**
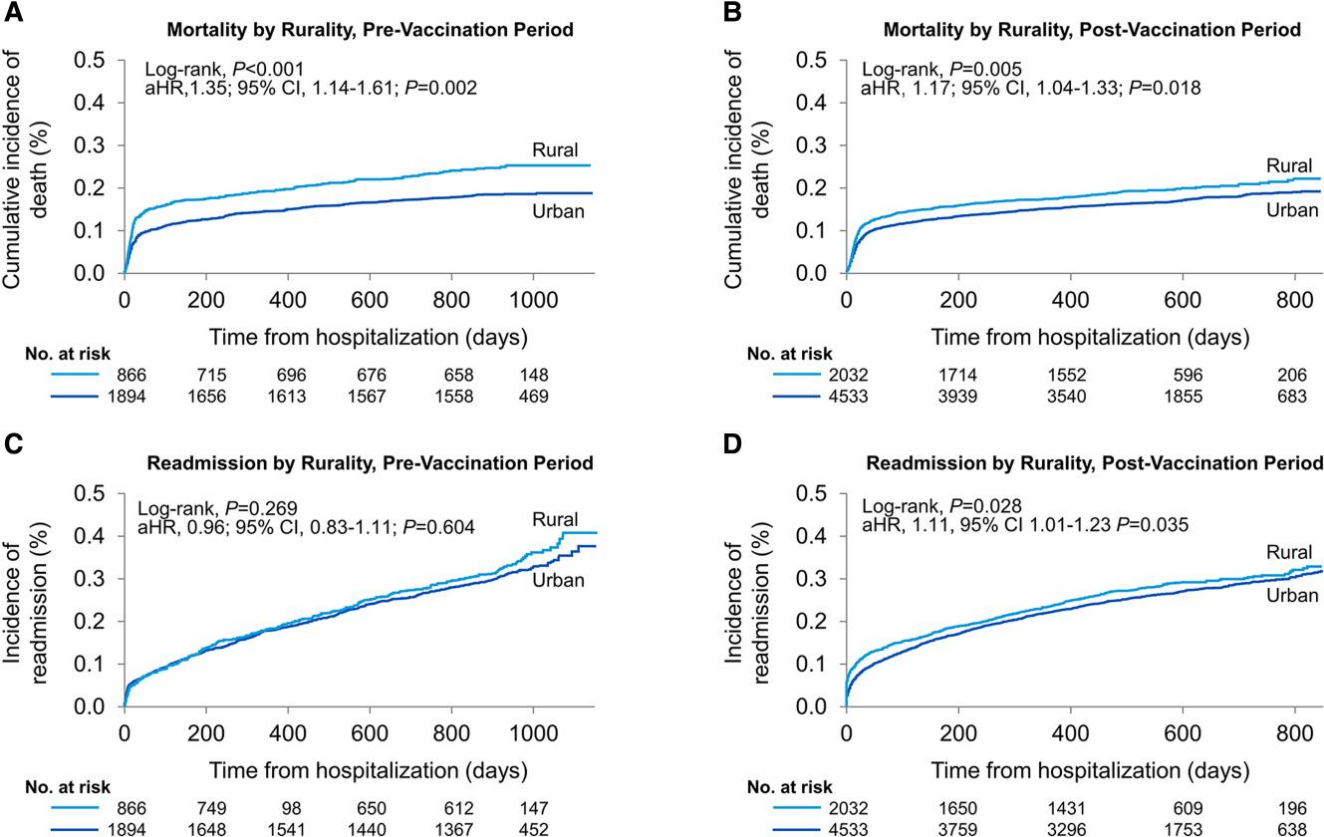
Kaplan-Meier estimates for mortality and readmission following COVID-19 hospitalization stratified by rural and urban county of residence. *A*, Prevaccination period: Kaplan-Meier estimates for mortality rates, categorized according to whether patients reside in rural or urban areas. *B*, Postvaccination period: Kaplan-Meier estimates for mortality rates, categorized according to whether patients reside in rural or urban areas. *C*, Prevaccination period: Kaplan-Meier estimates for readmission rates, categorized according to whether patients reside in rural or urban areas. *D*, Postvaccination period: Kaplan-Meier estimates for readmission rates, categorized according to whether patients reside in rural or urban area. Abbreviations: aHR, adjusted to hazard ratio; CI, confidence interval.

The prevaccination period comprised 2760 patients (30%) and the postvaccination period included 6565 patients (70%). Mortality and readmission rates were 568 (21%) and 893 (32%) in the prevaccination, and 1170 (18%) and 1836 (28%) in the postvaccination timeframes, respectively. A Cox proportional hazard model adjusted for 31 covariates demonstrated a 35% increase in mortality (log-rank *P* < .001; HR, 1.35; 95% CI, 1.14-1.61; *P* = .002) and a no change in readmission rate (log-rank *P* = .269; HR, 0.96; 95% CI, .83-1.11; *P* = .604) for rural compared with urban residents in the prevaccination timeframe (Figure 4). Similarly, in the postvaccination timeframe, there was a 17% increase in mortality (log-rank *P* = .005; HR, 1.17; 95% CI, 1.04-1.33; *P* = .018) and a 11% increase in readmission rate (log-rank *P* = .028; HR, 1.11; 95% CI, 1.01-1.23; *P* = .035) for rural residents.

The STROBE check list is provided in Supplementary Table 5.

## DISCUSSION

This multicenter study of the patients hospitalized for COVID-19 between March 2020 and July 2022 (followed through May 2023) with longer follow-up than previously reported [7–10, 15, 16] highlights several novel findings. First, rurality emerged as a strong predictor of higher long-term risk of death independent of 31 covariates from multiple domains, which were previously known to impact COVID-19 outcomes [6, 16–22]. Patients living in rural areas, as opposed to urban areas, experienced a 4% absolute increase in mortality, indicating that rurality was linked to one additional death for every 25 patients hospitalized for COVID-19 at a median follow-up of 602 days. Second, the results of the sensitivity analysis reinforced findings from main analysis. Third, the higher mortality in rural versus urban patients, which was observed in the first year of the COVID-19 pandemic was maintained in subsequent pandemic years. Fourth, the rural-urban difference in mortality was, in part, accounted for by factors including age ≥65 years, race, and receiving JAK1/2 inhibitors/IL-6 inhibitors. Noticeably, White adults aged ≥65 years make up a disproportionate share of rural population in our study and previous reports in other geographical areas [23, 24]. Finally, the findings of increased mortality in rural compared with urban residents remained consistent across both pre- and postvaccination timeframes. Notably, the readmission rates following initial COVID-19 hospitalization demonstrated a trend toward being higher in rural residents compared with urban residents.

With a median follow-up of 602 days, our findings extend the results of prior research that examined COVID-19 hospitalization outcomes up to 1 year [4, 5, 18, 25, 26]. Despite enactment of public health measures to reduce the disparity in healthcare delivery [27] the large gap across the rural-urban continuum with worse outcomes in rural patient was potentially compounded by the dynamics of the COVID-19 pandemic. Our results were comparable to those of other acute conditions with similar troubling mortality trends across rural and urban patients [28]. During the pandemic, both mortality and readmission rates varied considerably with timeframe, levels of SARS-CoV-2 activity, geographical location, patient-level characteristics, and treatment strategies [22, 29, 30]. Mortality rates as high as 29% within 60 to 180 days of hospitalization for COVID-19 have been reported from Europe and early epicenters in the United States [7, 8, 31, 32]. These rates are substantially higher than more recent reports from the United States [33] and the findings of the current study, which showed an overall mortality of 19% within a 3.14-year follow-up period. With the exception of the early stages of the pandemic, short-term mortality rates attributed to COVID-19 have consistently been higher among rural than in urban residents [4, 5, 18, 25, 26]. These studies focused on shorter timeframes for mortality and were primarily conducted during the early stages of the pandemic, which limits direct comparisons with the current study [16, 34].

Similarly, there has been limited research investigating differences in readmission rates between rural and urban areas, particularly over the long term. Individual studies and meta-analyses reported overall readmission rates ranging from 3.6% to 10.4% within 3 to 180 days after the initial hospitalization for COVID-19, showing considerable geographic variation [22, 30, 35]. This contrasts with a 29% readmission rate at a median follow-up of 602 days in the main cohort of this study.

In this study, we analyzed baseline differences in sociodemographic factors, comorbidities, admission service, and the year of the pandemic, uncovering disparities in outcomes between rural and urban areas. We found that rural patients had higher rates of comorbidities such as obesity, heart failure, chronic obstruction pulmonary disease, depression, and substance use, which have been previously associated with poorer outcomes [6, 16–22]. Additionally, we observed that rural patients with COVID-19 were more likely to be admitted to critical care and pulmonary services compared with their urban counterparts, which was associated with poorer clinical outcomes. The rural population in the United States is one of the most vulnerable groups, often having limited access to high-quality postacute primary and specialty care [36, 37]. Technological advancements in healthcare delivery have had a minimal impact on rural patients. For example, telehealth services, which saw increased use during the COVID-19 pandemic, provided fewer benefits for rural populations [38]. The constraints in postacute care services may have contributed to higher long-term mortality rates among patients from rural counties, and this requires further investigation in future studies. Furthermore, consistent with prior studies, we observed that rural patients hospitalized for COVID-19 were older and had a higher burden of comorbidities compared with their urban counterparts, potentially influencing long-term survival [39]. The effect of age and the comorbidities on COVID-19 outcomes was identified early in the pandemic and has remained consistent throughout its duration [18]. Patients who were readmitted had a higher prevalence of baseline comorbidities compared with those who were not readmitted [16, 21, 22, 35]. With a median time to readmission exceeding 150 days, it is improbable that COVID-19 is the primary cause of most readmissions. This implies that other factors, such as the exacerbation of existing chronic conditions or the emergence of new health issues, may contribute to the longer-term need for readmission. Conversely, with a median time to mortality of 30 days, COVID-19 is likely a significant factor in all-cause deaths.

### Strength and Limitations

This study has several strengths, including minimal missing data, inclusion of laboratory-confirmed COVID-19 infections, hospital-level characteristics (admission service and receipt of EUA medications), a variety of chronic conditions not previously reported in other studies, and the comparison of pre- and postvaccination timeframes. The study captured COVID-19 hospitalizations during the predominant Alpha, Delta, early Omicron (January-March 2022), and late Omicron (April-June 2022) periods. Our multivariable-adjusted models accounted for several confounders, including sociodemographic factors, 24 chronic conditions, pandemic calendar year, admission service, and EUA-directed COVID-19 treatment. Most of the rural population of the study were from southeastern Minnesota, western Wisconsin, and northeastern Iowa, areas that share demographic characteristics with the wider Upper Midwest region of the United States [40, 41]. The findings from the sensitivity analysis, which focused on limited geographical areas where study sites were located, and subgroup analyses reinforced the results of the main analysis. Our results should be interpreted in the context of certain limitations. Patients admitted to hospitals in the integrated Mayo Clinic system originated from diverse counties across the United States; thus, our study may not have fully captured some of the clinical outcomes, especially readmissions to the study centers. To address the potential issue of incomplete outcome capture, we conducted a sensitivity analysis focusing on restricted geographical areas surrounding the study centers. The analysis restricted to states where the study centers were located suggests that the associations between rurality and outcomes after hospitalization for COVID-19 were robust.

## CONCLUSIONS

Patients from rural counties experienced higher long-term mortality rates following COVID-19 hospitalization compared with those from urban counties, a difference that continued despite the rollout of COVID-19 vaccines during the study period. The striking rural-urban difference in long-term mortality after COVID-19 hospitalization underscore the necessity for further efforts to develop comprehensive, multidisciplinary approaches to reduce these disparities.

## Supplementary Material

ofae197_Supplementary_Data

## Supplementary Data

Supplementary materials are available at *Open Forum Infectious Diseases* online. Consisting of data provided by the authors to benefit the reader, the posted materials are not copyedited and are the sole responsibility of the authors, so questions or comments should be addressed to the corresponding author.

## Note

***Potential conflicts of interest*.** All authors: No reported conflicts.
