# Supplementary material for: Rural-urban Differences in Long-term Mortality and Readmission Following COVID-19 Hospitalization, 2020 to 2023
Source: Open Forum Infect Dis. 2024 Apr 5;11(5):ofae197. doi: 10.1093/ofid/ofae197 (PMC11065360; doi:10.1093/ofid/ofae197)
Supplement: ofae197_Supplementary_Data [file ofae197_supplementary_data.pdf]

Rural-urban differences in Long-term Mortality and Readmission Following COVID-19 Hospitalization, 2020 to 2023

Supplement

Supplement Figure 1. STROBE flow diagram.

Supplement Table 1. Covariates for multivariable analysis

Supplement Table 2. ICD-10-CM codes for chronic disease conditions

Supplement Table 3. Correlation matrix

Supplement Table 4. Variance Inflation Factor (VIF) values for predictor variables

Supplement Figure 2. Logistic regression analysis in the main cohort

Supplement Figure 3. logistic regression analysis in the sensitivity cohort

Supplement Figure 4. Sub-group analysis in the sensitivity cohort.

Supplement Table 5. STROBE checklist.

Supplemental eFigure 1. Strengthening The Reporting of Observational studies in Epidemiology (STROBE) flow diagram for the selection of study cohorts.

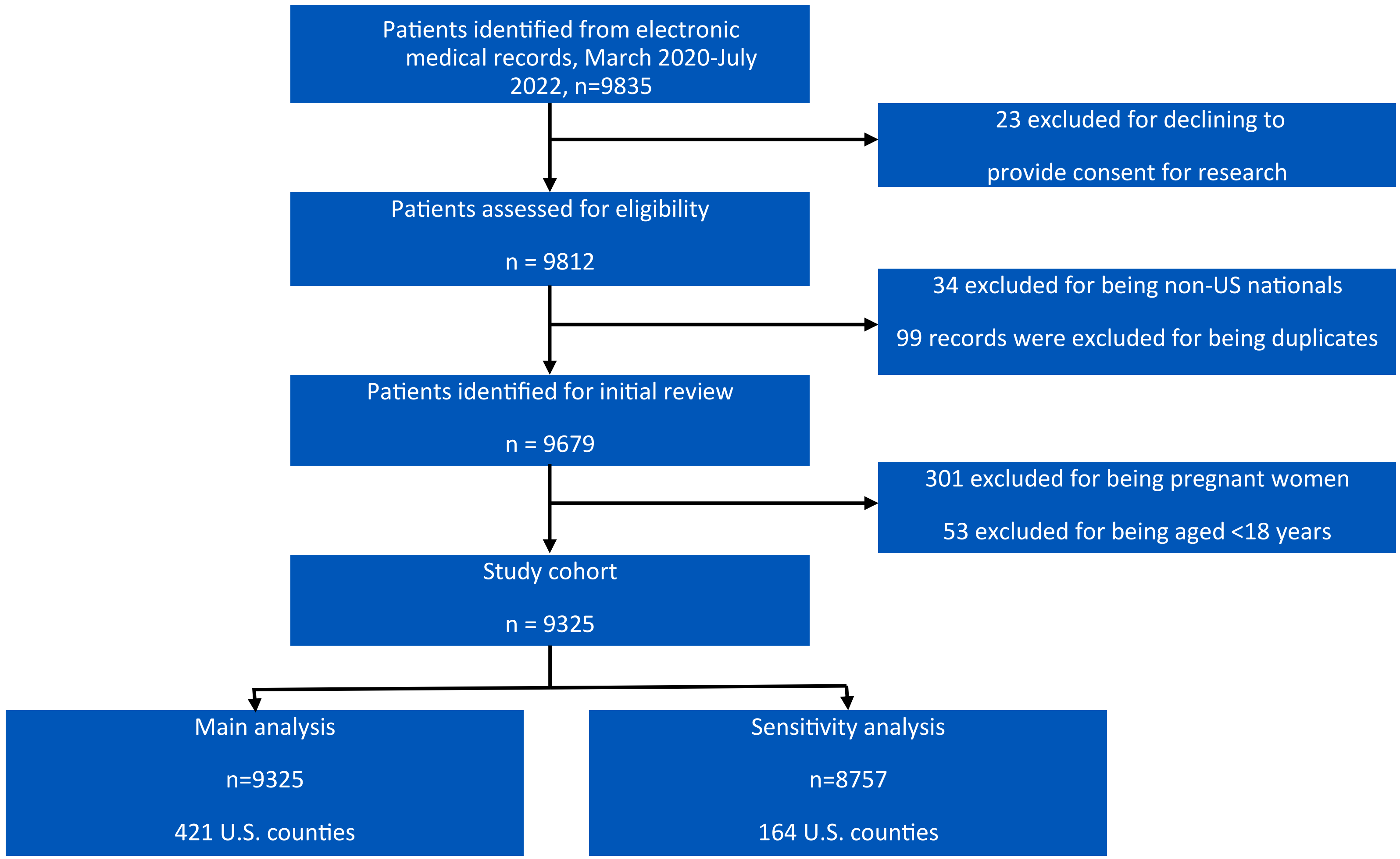

Supplement eTable 1. Covariates for multivariable analysis

**Demographics**	
Age, Sex at birth, Race (white or nonwhite)	
**Social indicators**	
Marital status (married or nonmarried), cigarette smoking (current smoking or no smoking)	
**Comorbidities**	
Cardiovascular:	Hypertension, coronary artery disease, atrial fibrillation, heart failure, stroke
Respiratory:	Asthma, chronic obstructive pulmonary disease
Endocrine and metabolism:	Hyperlipidemia, diabetes mellitus, obesity, malnutrition
Neurological:	Dementia
Liver:	All chronic liver diseases combined (cirrhosis, viral and autoimmune hepatitis, Hemochromatosis)
Kidney:	Chronic kidney disease
Hematology:	Anemia
Rheumatology:	Osteoarthritis
Oncology:	All cancers combined (solid organ and bone marrow)
Transplant & Immunology:	All transplants (solid organ, bone marrow, stem cell) and primary immune disorders combined
Psychiatric Diagnoses:	Depression (major depression and bipolar disorder), other combined chronic psychiatric condition (generalized anxiety disorder, post-traumatic stress disorder, panic disorder, schizophrenia), and substance use disorder
**Admission service**	
Specialty service:	Combined pulmonology and critical care
**Emergency Use Authorization COVID-19 treatment**	
Remdesivir, dexamethasone, Janus kinase1/2 inhibitors/ interleukin-6 inhibitor	
**Year of the COVID-19 pandemic**	
2020 (March – December), 2021 (January – December 2021), and 2022 (January – July 2022)	

Supplement eTable 2. ICD-10-CM codes for chronic disease conditions

Category	Description of individual chronic condition	ICD-10-CM codes
**Cardiovascular**	Hypertension	I10, I11.9, I15.0, I15.9
	Coronary artery disease	I20.0, I21.4, I21.AI, I25.42, I25.82, I25.110, I25.111, I125.118, I25.119, I25.710, Z95.1, Z95.5, Z98.61, Z98.61
	Atrial fibrillation or flutter	I48.0, I48.1, I48.11, I48.19, I48.2, I48.20, I48.3, I48.4, I48.91, I48.91
	Heart failure	
	Stroke	I61.11, I60.12, I60.31, I60.32, I60.4, I60.51, I60.6, I60.7, I60.8, I60.9, I61.1, I61.2, I61.5, I61.6, I61.8, I61.9, I62.00I63.00, I63.011, I63.012, I63.019, I63.02, I63.22, I63.233, I63.30, I63.031, I63.032, I63.10, I63.311, I63.20, I63.212, I63.219, I63.239, I63.321, I63.232, I63.312, I63.321, I63.322, I63.332, I63.331, I63.333, I63.39, I63.341, I63.342, I63.343, I63.413, I63.419, I63.429, I63.431, I63.432, I63.439, I63.40, I63.511, I63.512, I63.513, I63.521, I63.522, I63.523, I63.532, I63.541, I63.542, I63.543, I63.50, I63.59, I63.8, I63.81, I63.89, I63.9, I69.054, I69.122, I69.151, I69.154, I69.198, I69.222, I69.251, I69.254, I69.328, I69.331, I69.332, I69.331, I69.334, I69.352, I69.354, I69.359, I69.392, I69.398, I69.822, I69.851, I69.852, I69.854, I69.922, I69.928, I69.931, I69.934, I69.939, I69.951, I69.954, I69.959, I69.992, G81.90, G81.91, G81.92, G81.93, G81.94
**Respiratory**	Asthma	J45.20, J45.21, J45.30, J45.31, J45.40, J45.41, J45.50, J45.51, J45.901, J45.909
	COPD	J44.0, J44.1, J44.9
**Neurologic**	Dementia	F02.80, F02.81, F0.90, F0.91, F01.50, F0.51, G30.0, G30.1, G30.9, G31.09, G31.83,
**Endocrine and Metabolism**	Hyperlipidemia	E78.00, E78.2, E78.5
	Diabetes mellitus (Type 1 and Type 2)	E08.9, E08.10, E08.11, E08.21, E08.22, E08.40, E08.42, E08.52, E08.65, E08.649, E08.620, E08.3513, E08.3593, E10.9, E10.10, E10.11, E10.21, E10.22, E10.36, E10.40, E10.40, E10.43, E10.51, E10.52, E10.65, E10.69,E10.319, E10.621, E10.641, E10.649,E10.3513, E10.3593,
	Obesity	E66.01, E66.09, E66.09, E66.2, E66.8, E66.9, Z68.30, Z68.31, Z68.32, Z68.33, Z68.34, Z68.35,

		Z68.36, Z68.37, Z68.38, Z68.39, Z68.40, Z68.41, Z68.42, Z68.43, Z68.44, Z68.45
	Malnutrition	E43, E43.0, E44.0, R64,
**Liver disease**	All combined (cirrhosis, viral and autoimmune hepatitis, hemochromatosis)	B18.1, B18.2, B19.20, E83.111, E83.119, K70.2, K70.9, K70.10, K70.11, K70.30, K70.31, K70.40, K72.00, K72.01, K72.10, K72.90, K72.91, K74.00, K74.60, K74.69, K75.9, K75.81, K75.89, K76.7,
**Kidney**	Chronic kidney disease	N18.3, N18.31, N18.32, N18.4, N18.5, N18.6, Z99.2,
**Hematology**	Anemia	D50.0, D52.9, D63.0, D63.1, D63.8, D64.81, D64.9,
**Rheumatology**	Osteoarthritis	G61.9, M06.4, M10.9, M16.0, M17.0, M17.10, M17.11, M17.12
**Oncology**	All malignant conditions	C01, C02.1, C02.3, C02.9, C03.9, C06.1, C06.9, C07, C7A.00, C7A.010, C7A.012, C7A.019, C7A.020, C7A.025, C7A.090, C7A.092, C7A.098, C10.9, C11.9, C14.0, C15.4, C15.5. C15.8, C15.9, C16.0, C16.1, C16.2, C16.5, C16.6, C16.9, C17.2, C18.0, C18.1, C18.2, C18.3, C18.4, C18.5, C18.6, C18.7, C18.8, C18.9, C19, C20, C21.0, C21.1, C21.8, C22.0, C22.1, C22.9, C23, C24.0, C24.1, C24.9, C25.0, C25.1, C25.2, C25.4, C25.7, C25.9, C26.1, C26.9, C30.0, C32.1, C32.9, C34.01, C34.02, C34.10, C34.11, C34.12, C34.2, C34.30, C34.31, C34.32, C32.8, C34.80, C34.81, C34.82, C34.90, C34.91, C34.92, C37, C38.3, C41.1, C41.2, C41.4, C41.9, C47.9, C48.0, C48.2, C49.0, C49.21, C49.22, C49.3, C49.4, C49.8, C49.9, C50.012, C50.111, C50.112, C50.119,C50.211, C50.212, C50.312, C50.411, C50.412, C50.512, C50.811, C50.812, C50.819, C50.911, C50.912, C50.919, C51.0, C51.9, C53.0, C53.8, C53.9, C54.1, C55, C56.1, C56.2, C56.3, C56.9, C57.01, C57.7, C57.8, C57.9, C60.9, C61, C62.11, C64.1, C64.2, C64.9, C65.9, C66.1, C66.2, C66.9, C67.0, C67.1, C67.2, C67.4, C67.6, C67.8, C67.9, C68.8, C69.2, C69.21, C69.22, C69.31, C69.32, C70.1,C71.2, C71.3, C71.1, C71.6, C71.8, C71.9, C72.0, C73, C74.91, C74.92, C74.99, C75.9, C76.0, C76.1, C76.3, C76.42, C76.52, C76.8, C81.00, C81.02, C81.08, C81.10, C81.12, C81.18, C81.19, C81.90, C82.00, C82.02, C82.03, C82.04, C82.08, C82.09, C82.10, C82.11, C82.13, C82.15, C82.16, C82.17, C82.18, C82.19, C82.20, C82.21, C82.28, C82.29, C82.31, C82.38, C82.39, C82.40, C82.48, C82.49, C82.58, C82.80, C82.88, C82.89, C82.90, C82.93, C82.98, C82.99, C83.00, C83.01,

		C83.02, C83.03, C83.06, C83.07, C83.09, C83.10, C83.11, C83.12, C83.13, C83.18, C83.19, C83.30, C83.31, C83.32, C83.33, C83.34, C83.36, C83.37, C83.38, C83.39, C83.70, C83.73, C83.78, C83.79, C84.40, C84.48, C84.49, C84.60, C84.70, C84.71, C84.73, C84.74, C84.78, C84.79, C85.20, C85.22, C85.28, C85.29, C85.80, C85.82, C85.83, C85.88, C85.89, C85.90, C85.91, C85.92, C85.93, C85.94, C85.96, C85.98, C85.99, C86.0, C88.0, C88.4, C90.00, C90.02, C91.10, C91.11, C91.12, C91.40, C91.41, C91.Z0, C92.10, C92.11, C92.12, C92.50, C92.51, C92.90, C93.10, C93.12, C94.6, C95.00, C95.01, C95.02, C95.10, C95.90, D05.11, D45, D75.81, D47.1, R18.0
**Transplant and immunology**	All transplants (solid organ, bone marrow, stem cell) and primary immune disorders combined	D80.1, D80.2, D84.9, D84.81, D84.89, Z94.0, Z94.1, Z94.2, Z94.4, Z94.81, Z94.83, Z94.84
**Psychiatric conditions**	Depression (major depression and bipolar disorder)	F30.2, F30.3, F30.4, F30.9, F30.10, F30.12, F31.2, F31.4, F31.5, F31.9, F31.10, F31.12, F31.13, F31.30, F31.31, F31.32, F31.60, F31.62, F31.63, F31.64, F31.70, F31.71, F31.73, F31.74, F31.75 F31.76, F31.77, F31.81, F31.89, F32.1, F32.2, F33.0, F33.0, F33.1, F33.2, F33.3, F33.9, F33.40, F33.41, F33.42,
	other combined chronic psychiatric condition (generalized anxiety disorder, post-traumatic stress disorder, panic disorder, schizophrenia), panic disorder, schizophrenia),	F20.0, F20.9, F20.81, F21, F25.0, F25.1, F25.9, F40.10, F41.0, F41.1, F43.10, F43.12,

Supplement eTable 3. Correlation matrix for predictor variables

	age	sex	not_married_2	white_1	service_c	afib	hf	cad	ckd	obesity	liver	arthritis	dementia
age	1	-0.04	0	-0.12	-0.07	0.32	0.24	0.16	0.21	-0.23	-0.05	0.11	0.2
sex	-0.04	1	-0.15	0.04	0.04	0.06	0.01	0.08	0.03	-0.03	0.02	0.01	-0.02
not_married_2	0	-0.15	1	0.02	0	0.03	0.06	0	0.04	0	0.03	0	0.06
white_1	-0.12	0.04	0.02	1	0.05	-0.06	-0.04	-0.03	-0.03	-0.01	0.02	0.01	-0.04
service_cc_OR_service_specialty	-0.07	0.04	0	0.05	1	0.03	0	0	0	0.02	0.07	0	-0.04
afib	0.32	0.06	0.03	-0.06	0.03	1	0.35	0.12	0.2	-0.05	-0.01	0.08	0.05
hf	0.24	0.01	0.06	-0.04	0	0.35	1	0.2	0.27	0.02	0.01	0.09	0.04
cad	0.16	0.08	0	-0.03	0	0.12	0.2	1	0.14	-0.03	-0.01	0.04	0.02
ckd	0.21	0.03	0.04	-0.03	0	0.2	0.27	0.14	1	-0.04	0.02	0.11	0.03
obesity	-0.23	-0.03	0	-0.01	0.02	-0.05	0.02	-0.03	-0.04	1	-0.01	0.02	-0.1
liver	-0.05	0.02	0.03	0.02	0.07	-0.01	0.01	-0.01	0.02	-0.01	1	0.02	0.01
arthritis	0.11	0.01	0	0.01	0	0.08	0.09	0.04	0.11	0.02	0.02	1	0.02
dementia	0.2	-0.02	0.06	-0.04	-0.04	0.05	0.04	0.02	0.03	-0.1	0.01	0.02	1
hlp	0.21	0.04	-0.04	-0.01	-0.02	0.1	0.12	0.13	0.16	0.01	0.03	0.16	0.05
htn	0.19	0.01	-0.01	0.03	0.02	0.09	0.08	0.07	0.19	0.04	0.05	0.14	0.04
malnutrition	0.03	0.02	0.01	0.01	0.06	0.02	-0.01	-0.02	0.03	-0.11	0.1	0.06	0.05
depression	0.01	-0.14	0.09	-0.07	-0.03	0.01	0.08	0.04	0.07	0.06	0.02	0.06	0.03
psych	-0.08	-0.1	0.06	-0.03	0	-0.03	0.02	-0.01	-0.02	0.04	0.04	0.03	0.03
stroke	0.1	0.01	0.02	-0.01	0.02	0.08	0.06	0.06	0.08	-0.04	0.03	0.02	0.13
immune_transplant	-0.09	0	-0.02	0.03	0.12	-0.02	-0.03	0.01	0.11	-0.08	0.08	0.04	-0.03
anemia	0.11	-0.02	0.05	-0.01	0.1	0.1	0.14	0.06	0.22	-0.07	0.12	0.06	0.05
substance_use	-0.11	0.06	0.09	-0.01	0.06	0	0	-0.02	-0.03	0.01	0.22	0.01	-0.01
copd	0.17	0.01	0.07	-0.03	-0.01	0.12	0.17	0.07	0.1	-0.03	0.03	0.07	0.04
dm_all	0.04	0.03	0.02	0.07	0.05	0.04	0.08	0.06	0.17	0.11	0.05	0.09	0.01
cancer_all	0.1	0.03	-0.04	-0.02	0.02	0.04	0.01	0.01	0.04	-0.08	0.04	0.03	0.02
remdesivir	-0.1	0	0.02	-0.01	-0.03	-0.09	-0.07	-0.05	-0.17	0.05	0.01	-0.06	0.02
dexameth	-0.12	0.02	-0.02	-0.02	0.04	-0.08	-0.06	-0.05	-0.15	0.07	0.02	-0.05	-0.04
jak1/2inhibitor/IL-6 inhibitor	-0.12	0.06	-0.02	0.02	0.15	-0.07	-0.08	-0.06	-0.1	0.08	0.03	-0.03	-0.04
smoking_current	-0.09	0.04	0.11	0.01	0	-0.02	0.01	0.01	-0.01	0	0.06	-0.01	0.01

	hlp	htn	malnutrition	depression	psych	stroke	immune_	anemia	substanc	copd	dm_all	cancer_a	remdesiv	dexamet	jak1/2inl	smoking_
age	0.21	0.19	0.03	0.01	-0.08	0.1	-0.09	0.11	-0.11	0.17	0.04	0.1	-0.1	-0.12	-0.12	-0.09
sex	0.04	0.01	0.02	-0.14	-0.1	0.01	0	-0.02	0.06	0.01	0.03	0.03	0	0.02	0.06	0.04
not_married_2	-0.04	-0.01	0.01	0.09	0.06	0.02	-0.02	0.05	0.09	0.07	0.02	-0.04	0.02	-0.02	-0.02	0.11
white_1	-0.01	0.03	0.01	-0.07	-0.03	-0.01	0.03	-0.01	-0.01	-0.03	0.07	-0.02	-0.01	-0.02	0.02	0.01
service_cc_OR_service_specialty	-0.02	0.02	0.06	-0.03	0	0.02	0.12	0.1	0.06	-0.01	0.05	0.02	-0.03	0.04	0.15	0
afib	0.1	0.09	0.02	0.01	-0.03	0.08	-0.02	0.1	0	0.12	0.04	0.04	-0.09	-0.08	-0.07	-0.02
hf	0.12	0.08	-0.01	0.08	0.02	0.06	-0.03	0.14	0	0.17	0.08	0.01	-0.07	-0.06	-0.08	0.01
cad	0.13	0.07	-0.02	0.04	-0.01	0.06	0.01	0.06	-0.02	0.07	0.06	0.01	-0.05	-0.05	-0.06	0.01
ckd	0.16	0.19	0.03	0.07	-0.02	0.08	0.11	0.22	-0.03	0.1	0.17	0.04	-0.17	-0.15	-0.1	-0.01
obesity	0.01	0.04	-0.11	0.06	0.04	-0.04	-0.08	-0.07	0.01	-0.03	0.11	-0.08	0.05	0.07	0.08	0
liver	0.03	0.05	0.1	0.02	0.04	0.03	0.08	0.12	0.22	0.03	0.05	0.04	0.01	0.02	0.03	0.06
arthritis	0.16	0.14	0.06	0.06	0.03	0.02	0.04	0.06	0.01	0.07	0.09	0.03	-0.06	-0.05	-0.03	-0.01
dementia	0.05	0.04	0.05	0.03	0.03	0.13	-0.03	0.05	-0.01	0.04	0.01	0.02	0.02	-0.04	-0.04	0.01
hlp	1	0.48	0.09	0.09	-0.01	0.15	0.11	0.07	-0.02	0.19	0.39	0.08	-0.12	-0.1	-0.05	0.05
htn	0.48	1	0.11	0.08	-0.02	0.14	0.14	0.07	0	0.17	0.36	0.06	-0.21	-0.18	-0.07	0.05
malnutrition	0.09	0.11	1	0	0	0.07	0.15	0.16	0.05	0.05	0.08	0.13	0.05	0.06	0.08	0.06
depression	0.09	0.08	0	1	0.25	0.04	-0.02	0.08	0.1	0.09	0.04	0	-0.09	-0.09	-0.08	0.07
psych	-0.01	-0.02	0	0.25	1	-0.01	-0.01	0.06	0.1	0.04	-0.04	0	0	-0.02	-0.04	0.03
stroke	0.15	0.14	0.07	0.04	-0.01	1	0.03	0.1	0.03	0.07	0.1	0.05	0.02	-0.01	0	0.04
immune_transplant	0.11	0.14	0.15	-0.02	-0.01	0.03	1	0.17	-0.01	0	0.12	0.16	0.01	0.02	-0.01	-0.02
anemia	0.07	0.07	0.16	0.08	0.06	0.1	0.17	1	0.08	0.06	0.09	0.17	0.05	0.06	0.01	0.02
substance_use	-0.02	0	0.05	0.1	0.1	0.03	-0.01	0.08	1	0.06	-0.02	0.02	0.04	0.05	0.02	0.19
copd	0.19	0.17	0.05	0.09	0.04	0.07	0	0.06	0.06	1	0.1	0.06	-0.04	-0.01	-0.05	0.21
dm_all	0.39	0.36	0.08	0.04	-0.04	0.1	0.12	0.09	-0.02	0.1	1	0	-0.1	-0.09	0	0.02
cancer_all	0.08	0.06	0.13	0	0	0.05	0.16	0.17	0.02	0.06	0	1	0.06	0.05	-0.03	0.04
remdesivir	-0.12	-0.21	0.05	-0.09	0	0.02	0.01	0.05	0.04	-0.04	-0.1	0.06	1	0.58	0.29	0.04
dexameth	-0.1	-0.18	0.06	-0.09	-0.02	-0.01	0.02	0.06	0.05	-0.01	-0.09	0.05	0.58	1	0.33	0.02
jak1/2inhibitor/IL-6 inhibitor	-0.05	-0.07	0.08	-0.08	-0.04	0	-0.01	0.01	0.02	-0.05	0	-0.03	0.29	0.33	1	-0.03
smoking_current	0.05	0.05	0.06	0.07	0.03	0.04	-0.02	0.02	0.19	0.21	0.02	0.04	0.04	0.02	-0.03	1

Supplement eTable 4. Variance Inflation Factor (VIF) values for predictor variables

Variable	VIF Value
Age	1.464
Sex	1.088
Marital status	1.068
Race	1.038
Speciality service	1.073
Atrial Fibrillation	1.245
Heart Failure	1.274
CAD	1.081
CKD	1.232
Obesity	1.141
Liver	1.078
Arthritis	1.052
Dementia	1.075
Hyperlipidemia	1.496
Hypertension	1.475
Malnutrition	1.095
Depression	1.139
Psych	1.096
Stroke	1.064
Transplant	1.154
Anemia	1.182
Substance use	1.133
COPD	1.148
Diabetes	1.295
Cancer	1.089
Remedesivir	1.622
Dexamethazone	1.62
JAK/IL-6 inhibitor	1.204
Current smoking	1.121

Supplement eFigure 2. Multivariable logistic regression analysis comparing patients from rural and urban counties in the main cohort.

Categories	Characteristics	Main study cohort, n=9,325				
		Rural n=2898 (31%)	Urban n=6427 (69%)	Forest plot	OR (95% CI)	P value
**Comorbidities**						
Cardiovascular	Hypertension	1400 (48)	3211 (50)		0.92 (0.83 – 1.01)	0.058
	CAD	189 (7)	448 (7)		0.91 (0.76 – 1.18)	0.274
	Heart failure	425 (15)	825 (13)		1.14 (1.00 – 1.30)	0.043
	Atrial fibrillation	533 (18)	1065 (17)		1.13 (1.01 – 1.27)	0.030
	Stroke	164 (6)	299 (5)		1.21 (0.99 – 1.47)	0.057
Respiratory	Asthma	141 (5)	363 (6)		0.87 (0.71 – 1.06)	0.165
	COPD	423 (15)	669 (10)		1.45 (1.27 – 1.66)	<0.001
	OSA	587 (20)	1197 (19)		1.11 (0.99 – 1.24)	0.064
Endocrine & Metabolism	Hyperlipidemia	1213 (42)	2708 (42)		0.97 (0.88 – 1.06)	0.485
	Diabetes mellitus	883 (30)	1821 (28)		1.11 (1.01 – 1.22)	0.031
	Obesity	1628 (56)	3017 (47)		1.51 (1.38 – 1.66)	<0.001
	Malnutrition	256 (9)	731 (11)		0.75 (0.64 – 0.87)	0.001
Hepatology	Chronic liver disease	77 (3)	211 (3)		0.82 (0.63 – 1.68)	0.140
Kidney	CKD ≥ 3	464 (16)	935 (15)		1.10 (0.97 – 1.24)	0.145
Rheumatology	Osteoarthritis	244 (8)	578 (9)		0.92 (0.78 – 1.07)	0.277
Neurology	Dementia	108 (4)	280 (4)		0.83 (0.65 –1.04)	0.102
Hematology	Anemia	825 (28)	1844 (29)		0.98 (0.89 – 1.08)	0.670
Oncology	Cancer	358 (12)	891 (14)		0.84 (0.70 – 0.99)	0.049
Immunology	Immune deficiency	88 (3)	316 (5)		0.62 (0.49 – 0.79)	<0.001
Psychiatry	Major depression	449 (15)	795 (12)		1.29 (1.14 – 1.46)	<0.001
	Other psychiatric conditions	240 (8)	523 98)		1.03 (0.88 – 1.21)	0.719
	Substance use disorder	179 (6)	333 (5)		1.25 (1.03 – 1.50)	0.023
**Hospital-level characteristics**						
Admission service	Internal & Family medicine	2438 (84)	5735 (89)		0.63 (0.55 – 0.71)	<0.001
	Pulmonary & critical care	460 (16)	692 (11)		1.60 (1.41 – 1.82)	<0.001
Drug treatment	Remdesivir	1763 (61)	3592 (56)		1.24 (1.13 – 1.34)	<0.001
	Dexamethasone	1800 (62)	3777 (59)		1.16 (1.06 – 1.27)	0.001
	JAK1/2 inhibitors, IL-6 inhibitor	503 (17)	972 (15)		1.21 (1.07 – 1.36)	0.002

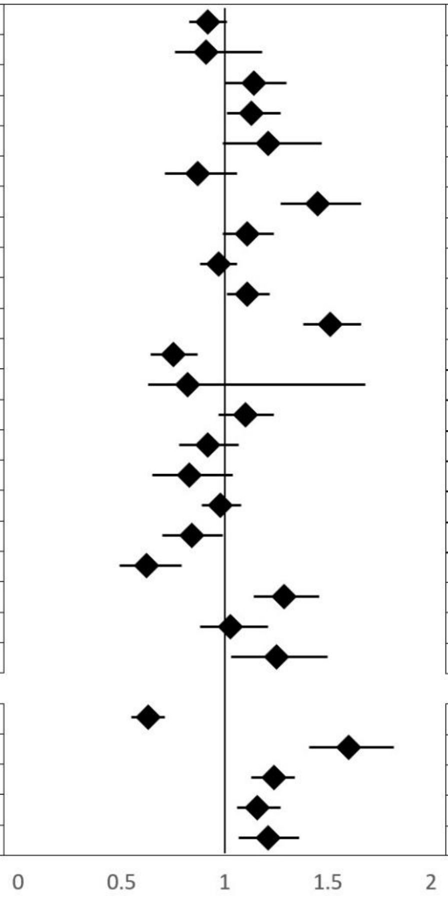

Caption: Comparative multivariable logistic regression analysis of patient- and hospital-level characteristics between rural and urban

residents in the main cohort presented as a forest plot with point estimates in odds ratio (OR) and corresponding 95% confidence intervals (CI) for the distribution of comorbidities and receipt of emergency use authorized drug treatment (EUA). Logistic regression models were adjusted to age, sex, race, and marital status.

Abbreviation: CAD, coronary artery disease; CKD, chronic kidney disease; COPD, chronic obstructive pulmonary disease; IL, interleukin; JAK, Janus kinase.

Supplement eFigure 3. Results of comparative multivariable logistic regression analysis of patient- and hospital-level characteristics between rural and urban residents in sensitivity analysis presented as a forest plot with point estimates in odds ratio (OR) and corresponding 95% confidence intervals (CI) for the distribution of comorbidities and receipt of emergency use authorized drug treatment (EUA).

Categories by System	Characteristic	Rural n=2607 (30%)	Urban n=6150 (70%)	Difference (%)	OR (95% CI)
Cardiovascular conditions	Hypertension	1,241 (48)	3,066 (50)	-2	0.90 (0.82-0.98)
	CAD	172 (7)	430 (7)	0	0.91 (0.75-1.09)
	Heart failure	394 (15)	793 (13)	2	1.16 (1.01-1.33)
	Atrial fibrillation	473 (18)	1,024 (17)	1	1.07 (0.94-1.22)
	Stroke	145 (6)	287 (5)	1	1.17 (0.95-1.44)
Respiratory	Asthma	128 (5)	352 (6)	-1	0.87 (0.71-1.08)
	COPD	386 (15)	649 (11)	4	1.43 (1.25-1.64)
Endocrine and metabolism	Hyperlipidemia	1,094 (42)	2,577 (42)	0	0.99 (0.90-1.09)
	Diabetes mellitus	792 (30)	1,744 (28)	2	1.10 (0.99-1.22)
	Obesity	1,480 (57)	2,898 (47)	10	1.55 (1.41-1.70)
	Malnutrition	217 (8)	688 (11)	-3	0.71 (0.61-0.84)
Liver disease	Chronic liver disease	73 (3)	203 (3)	0	0.85 (0.64-1.11)
Kidney disease	CKD ≥ 3	412 (16)	895 (15)	1	1.06 (0.93-1.21)
Rheumatology	Osteoarthritis	211 (8)	559 (9)	-1	0.87 (0.74-1.03)
Neurological conditions	Dementia	103 (4)	275 (4)	0	0.85 (0.67-1.08)
Hematology	Anemia	730 (28)	1,755 (29)	-1	0.95 (0.86-1.05)
Oncology	Cancer	304 (12)	832 (14)	-2	0.83 (0.72-0.96)
Transplant and immunology	Immune deficiency and organ transplant	106 (4)	510 (8)	-4	0.48 (0.39-0.59)
Chronic psychiatric conditions	Major depression	416 (16)	780 (13)	3	1.29 (1.13-1.17)
	Other psychiatric conditions	224 (9)	502 (8)	1	1.05 (0.89-1.24)
	Substance use disorder	161 (6)	325 (5)	1	1.17 (0.96-1.43)
EUA drug treatment	Remdesivir	1,611 (62)	3,445 (56)	8	1.28 (1.67-1.41)
	Dexamethasone	1,642 (63)	3,621 (59)	4	1.21 (1.10-1.33)
	JAK1/2 inhibitors/IL-6 inhibitor	460 (18)	932 (15)	3	1.23 (1.09-1.40)

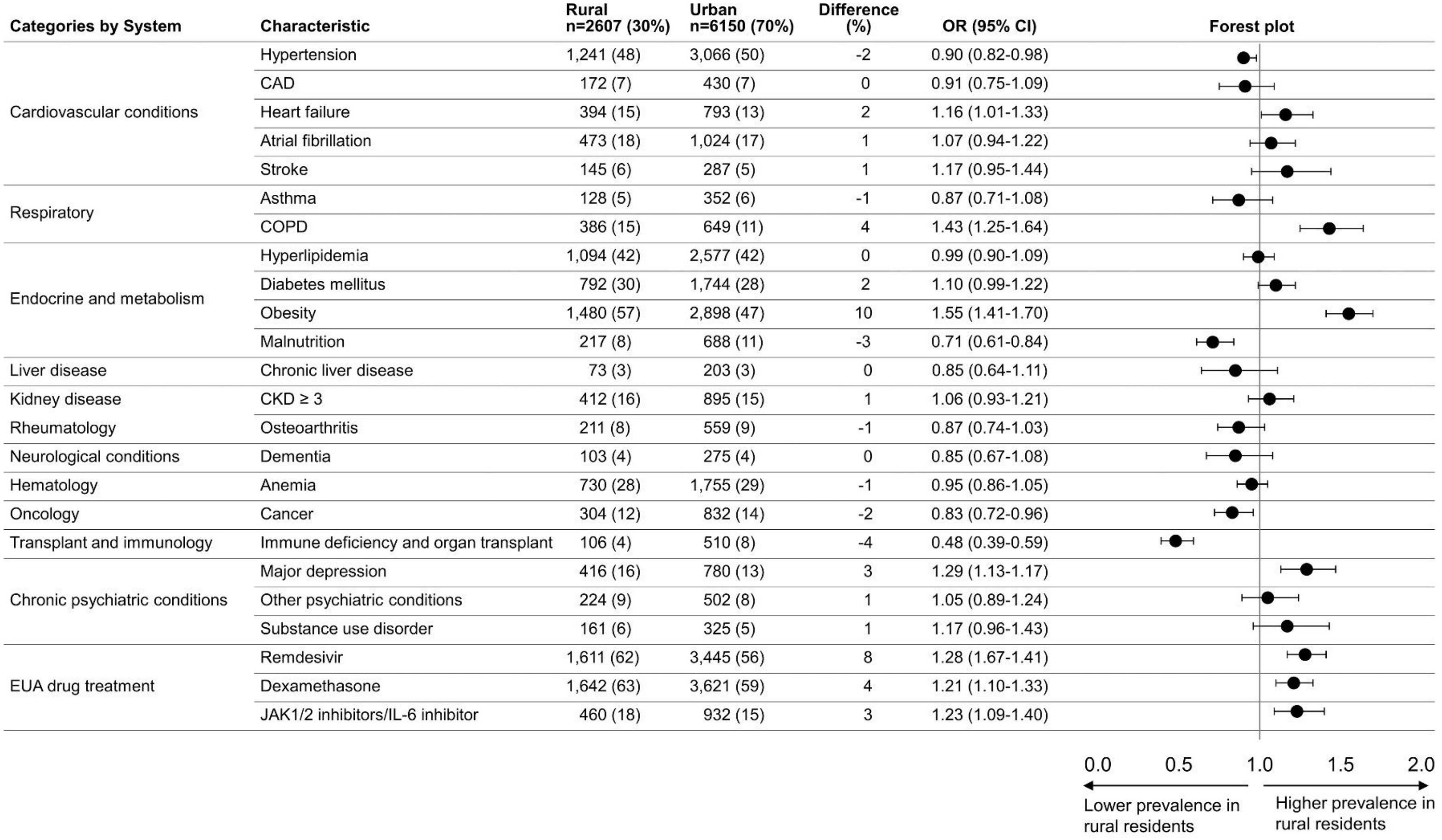

Logistic regression models were adjusted to age, sex, race, and marital status. Abbreviation: CAD, coronary artery disease; CKD, chronic kidney disease; COPD, chronic obstructive pulmonary disease; IL, interleukin; JAK, Janus kinase.

Supplement Figure 4. Sub-group analysis using Cox proportional hazard models in the sensitivity cohort (n=8757).

Categories	Subgroups	Rural event rate n = (%)	Urban event rate n = (%)	Forest plot rural vs. urban	HR (95% CI)	P value
**All-cause Mortality**						
All patients	All patients, n=8757	550 (21)	1076 (17)		1.25 (1.12-1.38)	<0.001
Age	<65 years, n=4132	115 (10)	244 (8)		1.03 (0.82-1.30)	0.796
	≥ 65 years, n=4625	435 (31)	832 (26)		1.29 (1.14-1.45)	<0.001
Sex	Female, n=3664	209 (19)	427 (17)		1.24 (1.05-1.47)	0.014
	Male, n=5093	341 (22)	649 (18)		1.25 (1.09-1.43)	0.0011
Race	White, n=7516	491 (22)	941 (20)		1.27 (1.13-1.42)	<0.001
	Nonwhite, n=1241	59 (17)	135 (15)		1.12 (0.81-1.55)	0.483
Marital status	Married, n=5021	264 (19)	561 (16)		1.17 (1.01-1.36)	0.039
	Nonmarried, n= 3736	286 (24)	515 (20)		1.30 (1.13-1.52)	0.001
Admission service	Specialties	129 (34)	231 (36)		0.98 (0.78-1.23)	0.858
	Non-specialties	421 (19)	845 (15)		1.34 (1.19-1.51)	<0.001
Emergency Use Authorization Drugs	Remdesivir, n=5056	297 (18)	549 (16)		1.18 (1.02-1.37)	0.024
	No remdesivir, n=3701	253 (25)	527 (19)		1.35 (1.16-1.58)	<0.001
	Dexamethasone, n=5263	330 (20)	644 (18)		1.15 (0.99-1.31)	0.052
	No dexamethasone, n=3494	220 (23)	432 (17)		1.45 (1.23-1.71)	<0.001
	JAK1/2 inhibitors/ IL-6 inhibitor, n=1392	117 (25)	241 (26)		0.97 (0.77-1.23)	0.823
	No JAK1/2 inhibitors/ IL-6 inhibitor, n=7365	433 (20)	835 (16)		1.35 (1.20-1.52)	<0.001
**All-cause readmission rate**						
All patients	All patients, n=8757	809 (31)	1788 (29)		1.10 (1.01-1.20)	0.024
Age	<65 years, n=4132	280 (23)	639 (21)		1.10 (0.95-1.27)	0.186
	≥ 65 years, n=4625	529 (38)	1149 (36)		1.09 (0.98-1.21)	0.105
Sex	Female, n=3664	342 (31)	776 (30)		1.06 (0.93-1.20)	0.396
	Male, n=5093	467 (31)	1012 (28)		1.13 (1.01-1.26)	0.027
Race	White, n=7516	725 (32)	1590 (30)		1.10 (1.01-1.20)	0.034
	Nonwhite, n=1241	84 (25)	198 (22)		1.09 (0.84-1.43)	0.514
Marital status	Married, n=5021	395 (28)	965 (27)		1.08 (0.96-1.21)	0.216
	Nonmarried, n= 3736	414 (35)	823 (32)		1.12 (1.00-1.27)	0.049
Admission service	Specialties, n=1023 (12)	81 (21)	179 (28)		0.81 (0.61-1.06)	0.121
	Non-specialties, n=7734 (88)	728 (33)	1609 (29)		1.14 (1.04-1.24)	0.005
Emergency Use Authorization Drugs	Remdesivir, n=5355	486 (30)	919 (27)		1.21 (1.08-1.36)	0.001
	No remdesivir, n=3970	323 (32)	869 (32)		0.96 (0.85-1.10)	0.588
	Dexamethasone, n=5577	494 (30)	995 (27)		1.20 (1.08-1.34)	0.001
	No dexamethasone, n=3748	315 (33)	793 (31)		0.97 (0.85-1.11)	0.672
	JAK1/2 inhibitors/ IL-6 inhibitor, n=1475	112 (24)	173 (19)		1.61 (1.26-2.07)	0.001
	No JAK1/2 inhibitors/ IL-6 inhibitor, n=7850	697 (32)	1615 (31)		1.05 (0.96-1.15)	0.301

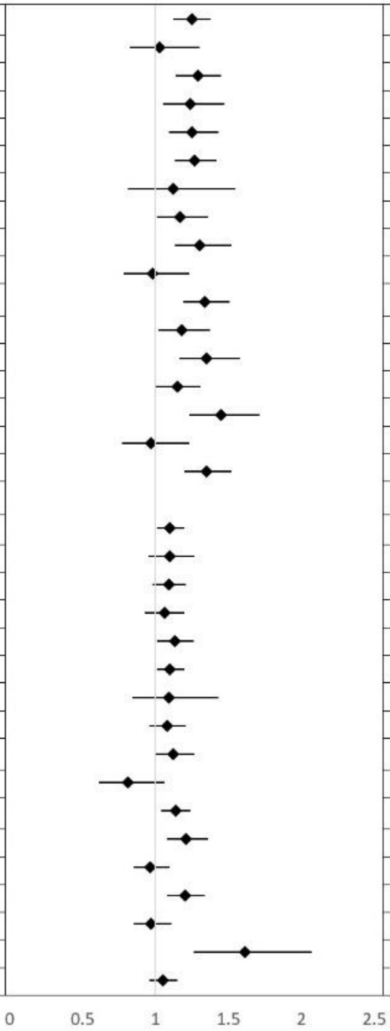

Supplement eTable 4.

STROBE Statement—checklist of items that should be included in reports of observational studies.

	Item No.	Recommendation	Page No.
**Title and abstract**	1	(*a*) Indicate the study's design with a commonly used term in the title or the abstract	2
		(*b*) Provide in the abstract an informative and balanced summary of what was done and what was found	2
**Introduction**			
Background/rationale	2	Explain the scientific background and rationale for the investigation being reported	5
Objectives	3	State specific objectives, including any prespecified hypotheses	5
**Methods**			
Study design	4	Present key elements of study design early in the paper	5-8
Setting	5	Describe the setting, locations, and relevant dates, including periods of recruitment, exposure, follow-up, and data collection	5-8
Participants	6	(*a*) *Cohort study*—Give the eligibility criteria, and the sources and methods of selection of participants. Describe methods of follow-up<br>*Case-control study*—Give the eligibility criteria, and the sources and methods of case ascertainment and control selection. Give the rationale for the choice of cases and controls<br>*Cross-sectional study*—Give the eligibility criteria, and the sources and methods of selection of participants	5-8
		(*b*) *Cohort study*—For matched studies, give matching criteria and number of exposed and unexposed<br>*Case-control study*—For matched studies, give matching criteria and the number of controls per case	

Variables	7	Clearly define all outcomes, exposures, predictors, potential confounders, and effect modifiers. Give diagnostic criteria, if applicable	6, 7
Data sources/ measurement	8*	For each variable of interest, give sources of data and details of methods of assessment (measurement). Describe comparability of assessment methods if there is more than one group	5-8
Bias	9	Describe any efforts to address potential sources of bias	n/a
Study size	10	Explain how the study size was arrived at	Retrospective study. No need for power calculation

Continued on next page

Quantitative variables	11	Explain how quantitative variables were handled in the analyses. If applicable, describe which groupings were chosen and why	6
Statistical methods	12	(*a*) Describe all statistical methods, including those used to control for confounding	7, 8
		(*b*) Describe any methods used to examine subgroups and interactions	
		(*c*) Explain how missing data were addressed	
		(*d*) *Cohort study*—If applicable, explain how loss to follow-up was addressed<br>*Case-control study*—If applicable, explain how matching of cases and controls was addressed<br>*Cross-sectional study*—If applicable, describe analytical methods taking account of sampling strategy	
		(*e*) Describe any sensitivity analyses	
**Results**			
Participants	13*	(a) Report numbers of individuals at each stage of study—eg numbers potentially eligible, examined for eligibility, confirmed eligible, included in the study, completing follow-up, and analysed	9-11
		(b) Give reasons for non-participation at each stage	
		(c) Consider use of a flow diagram	
Descriptive data	14*	(a) Give characteristics of study participants (eg demographic, clinical, social) and information on exposures and potential confounders	9-11
		(b) Indicate number of participants with missing data for each variable of interest	
		(c) *Cohort study*—Summarise follow-up time (eg, average and total amount)	
Outcome data	15*	*Cohort study*—Report numbers of outcome events or summary measures over time	9-10
		Case-control study—Report numbers in each exposure category, or summary measures of exposure	

		Cross-sectional study—Report numbers of outcome events or summary measures	
Main results	16	(*a*) Give unadjusted estimates and, if applicable, confounder-adjusted estimates and their precision (eg, 95% confidence interval). Make clear which confounders were adjusted for and why they were included	9-10
		(*b*) Report category boundaries when continuous variables were categorized	
		(*c*) If relevant, consider translating estimates of relative risk into absolute risk for a meaningful time period	

Continued on next page

Other analyses	17	Report other analyses done—eg analyses of subgroups and interactions, and sensitivity analyses	11
**Discussion**			
Key results	18	Summarise key results with reference to study objectives	11-12
Limitations	19	Discuss limitations of the study, taking into account sources of potential bias or imprecision. Discuss both direction and magnitude of any potential bias	13, 14
Interpretation	20	Give a cautious overall interpretation of results considering objectives, limitations, multiplicity of analyses, results from similar studies, and other relevant evidence	11-14
Generalisability	21	Discuss the generalisability (external validity) of the study results	11-14
**Other information**			
Funding	22	Give the source of funding and the role of the funders for the present study and, if applicable, for the original study on which the present article is based	15

*Give information separately for cases and controls in case-control studies and, if applicable, for exposed and unexposed groups in cohort and cross-sectional studies.

Note: An Explanation and Elaboration article discusses each checklist item and gives methodological background and published examples of transparent reporting. The STROBE checklist is best used in conjunction with this article (freely available on the Web sites of PLoS Medicine at http://www.plosmedicine.org/, Annals of Internal Medicine at http://www.annals.org/, and Epidemiology at http://www.epidem.com/). Information on the STROBE Initiative is available at www.strobe-statemen
